# Some thoughts on the possible health effects of electric and magnetic fields and exposure guidelines

**DOI:** 10.3389/fpubh.2022.994758

**Published:** 2022-09-15

**Authors:** Frank Barnes, Jr Eugene R. Freeman

**Affiliations:** Electrical Computer and Energy Engineering Department, University of Colorado, Boulder, CO, United States

**Keywords:** health effects, radio frequencies, ELF, magnetic fields, EM exposure

## Abstract

Concerns about the possible health effects from exposure to weak electric and magnetic (EM) fields have been debated since the early 1960s. It is now well established that biological systems respond to exposure to weak EM fields at energy levels well below the current safety guidelines which result in modification of their functionality without significant changes in temperature. These observations are adding to the debate over what should be done to protect the users of cellular telecommunications systems. Experimental results showing both increases and decreases in cancer cell growth rates and concentration of reactive oxygen species for exposure to nano-Tesla magnetic fields at both radio frequencies (RF) and extra low frequencies (ELF) are cited in this paper. Some theoretical models on how variations in EM exposure can lead to different biological outcomes and how feedback and repair processes often mitigate potential health effects due to long-term exposure to low-level EM energy sources are presented. Of particular interest are the application of the radical pair mechanisms that affect polarization of electrons, and nuclear spins and the importance of time-delayed feedback loops and the timing of perturbations to oscillations in biological systems. These models help account for some of the apparently conflicting experimental results reported and suggest further investigation. These observations are discussed with particular emphasis on setting future safety guidelines for exposure to electromagnetic fields in cellular telecommunications systems. The papers cited are a very small fraction of those in the literature showing both biological effects and no effects from weak electric and magnetic fields.

## Introduction

Concerns about possible health effects of exposure to EM fields date back to the early 1960's, especially regarding the impact of high energy radar systems and the effects of low frequency AC electric fields on people living near high voltage power lines. The initial concerns were associated with heating and shocks at high power densities and large electric fields. However, there have been continuing questions about possible effects at lower electric and magnetic field levels.

As radio frequency (RF) cellular telephony systems have expanded as the primary communication system in modern economies, concerns have grown regarding the effects of RF fields emitted by hand-held devices, WiFi stations and cell tower antennas. Most of these concerns are now focused on the pervasive long-term use of cell phones by large numbers of people and the introduction of 5G wireless systems that operate at higher frequencies and the increased number of cell phone towers. Considerable debate persists regarding the extent to which cell phone users should be protected from potential harm. Part of the debate centers on whether government regulations should lower the limits on device emissions is the result of the IARC classification of both ELF magnetic fields and RF as a possibly carcinogenic to humans' (1, 2).

The central problems to be solved include:

1. Determining the exposure amplitude, polarization and duration of electromagnetic fields at specific positions of interest in the body as a function of the exposing fields as a function of time.
2. Determining causal relationships between long duration exposure to low-level EM fields and the various biological responses reported in current research.
3. Quantifying the interactions between EM fields and biology with measurements that are not only accurate but repeatable. Some of the low energy EM effects on micro-biological systems are well documented in laboratory experiments but are difficult to translate to macro-biological system responses.
4. Sorting out which aspects of biological systems are directly driven by RF fields vs. the myriad of other independent variables at work.
5. Understanding how the repair mechanisms in biological systems alter biological processes in the presence of low-level EM fields.

A primary goal for scientists is to identify how the EM field driven effects on micro biological systems (cells, tissue samples, etc.) translate to outcomes in macro biological systems (brains, organs, tumors). The biggest challenge is to explain those processes and outcomes to a diverse assortment of scientific experts that rarely agree on “New Science” that might contradict established “Beliefs.”

When and if agreement in the scientific community is achieved, the central dilemma for regulators is to set operational standards that minimize harm to the users by reducing (or eliminating) the sources of harmful exposure. Such standards need to be based on quantifiable metrics for frequency, power, and duration of EM exposure etc. Codifying those standards and then deploying them in a manner that minimizes economic and social hardship is hard work. The biggest problem in all regulatory actions is how to enforce such measures when and if they are imposed on a user community that is already deeply dependent on RF transmission technology.

Several issues confront regulators:

1. Should regulations be set that “protect” all the population all the time, including those with other health conditions that make them more susceptible to EM exposure, or should regulations only protect most of the population most of the time. The degree of control has huge implications on cost and efficacy.
2. Simplifying the explanations of the physical mechanisms involved such that there is general acceptance of the need for regulation is a non-trivial part of the regulatory process. Scientists, producers, operators, and users have differing imperatives that need to be considered. Such situations invite a lot of political hubris and conflict.
3. How regulators quantify and then rationalize the tradeoffs between the economic and social benefits of cellular technology vs. potentially damaging health effects of long-term exposure to low levels of EM energy is important in determining the regulations that are proposed.

The lack of understanding and agreement in the scientific community on the physics, chemistry, and biology on the effects of exposure to low-level EM fields makes regulatory action nearly impossible at this point in time. The phenomena cross several scientific disciplines and require understanding and acceptance of how the linkages between electromagnetic fields at an atomic level due to electron and nuclear spins affect the chemistry of the human cell in ways that lead to problems with human health. Traversing these multiple disciplines not only requires credible data and rational theory on the cause-and-effect relationships, but also requires expertise from multiple disciplines that typically do not intersect in the scientific community. This paper attempts to create some bridges to close a few of the information and language gaps.

## Literature summary

There are important uses of electromagnetic fields in medicine and examples of both positive and negative health effects abound. In this paper we will cite only a small fraction of the work that shows biological systems can detect and respond to exposures to EM fields at power levels that are well below the current safety guidelines for cellular communications devices. We have placed emphasis on those where we have direct personal knowledge of the results (3) references indicating effects from weak magnetic fields and there are thousands of papers where no significant effects have been observed.

Furthermore, data indicate that long-term exposures to low levels of RF power can lead to effects that are cumulative and generally not seen for short-term exposures.

Among the experiments showing that biological systems can sense weak magnetic fields are those that show that birds and other animals can use the earth's magnetic field as an aid to navigation (4). Wang et al. (5) have shown that EEG signals in the human brain can be modified by magnetic fields as weak as 1 nT. Desai et al. (6) review a number of papers on the effects of RF radiation on male fertility that show significant damage for exposures to cell phone radiation and other wireless devices. McCormick (7) reviews many studies on the toxicity and potential oncogenicity at extremely low frequency magnetic fields in laboratory animal models that show both some indications of the induction of cancer, but that provide no compelling evidence of either significant chronic toxicity or oncogenicity of EMF at low frequencies in any organ. At RF frequencies the results are less conclusive (8). However, a large national toxicology study indicated a statistically significant increase in a cancer in male rat (9) and left open many questions. Static magnetic fields at 300 and 400 μT are shown to accelerate HT-1080 fibrosarcoma cancer cell growth rates and modify the concentrations of reactive oxygen species (ROS) with respect to those that are growing at 45 μT over periods of 4 days. These growth rates are inhibited at 0.5 μT and at 600 μT (10). Van Huizen et al. (11) showed static magnetic fields at 200 μT inhibit the regeneration rates in planarian and those at 500 μT accelerate them with respect to exposures at 45 μT. A more complete review of the literature prior to 2017 of the effects of static magnetic fields is given by Wang and Zhang (12). Novikov et al. (13, 14) showed that pulsed magnetic fields modify the concentrations of reactive oxygen species in neutrophils. Data on ELF effects are given in (15). Tofani (16) describes a possible link between weak magnetic fields, changes in reactive oxygen species and cancers and these weak magnetic fields as possible use in treating them. Usselman et al. (17, 18) show radio frequency magnetic fields of 10 μT rms at 7 MHz and a static field of 45 μT decreases the concentrations of superoxide, $O_{2}^{-}$, and increases hydrogen peroxide H_2_O_2_.

Reactive oxygen species such as H_2_O_2_ and NO are both signaling molecules and do damage when the concentrations are outside of their normal operating concentrations for extended periods of time (19, 20). Halliwell and Gutteridge (21) show the importance of controlling the concentrations of radicals and H_2_O_2_ in controlling biological processes to keep them in the normal operating range. Pooam et al. (22) show that exposures at 1 GHz for 15 min can modulate ROS in human HEK293 cells as a function of signal amplitude, changed gene expression and anti-oxidative enzymes (SOD, GPX, GPX, and CAT) and oxidative (Nox-2) enzyme concentrations. Responses are non-linear in amplitude and are frequency dependent. The results may be either increases or decreases and be harmful or beneficial.

Epidemiological studies on exposures to electromagnetic waves from cell phones and cell phone towers show mixed results with respect to possible health effects. The Interphone study (23, 24) report a reduced odds ratio (OR) related to ever having been a regular mobile phone user for glioma [OR 0.81; 95% confidence interval (CI) 0.70–0.94] and meningioma (OR 0.79; 95%CI 0.68–0.91), possibly reflecting participation bias or other methodological limitations. In the 10^th^ decile of recalled cumulative call time, > 1,640 h, the OR was 1.40 (95% CI 1.03–1.89) for glioma, and 1.15 (95% CI 0.81–1.62) for meningioma; but there are implausible values of reported use in this group. Zothansiama et al. (25) report exposed group (*n* = 40), residing within a perimeter of 80 m of mobile base stations, showed significantly (*p* <0.0001) higher frequency of micronuclei when compared to the control group, residing 300 m away from the mobile base stations. The analysis of various antioxidants in the plasma of exposed individuals revealed a significant attrition in glutathione (GSH) concentration (*p* <0.01), activities of catalase (CAT) (*p* <0.001) and superoxide dismutase (SOD) (*p* <0.001) and rise in lipid peroxidation (LPO) when compared to controls. There are other epidemiology studies that show increased odds ratios in the range from 1.5 to 2 for incidence of cancers after exposures to RF (26) and low frequencies. Kheifets et al. (27) as well as studies that indicate that there are no significant damaging effects.

To place this data in context the combined number of new cases of brain and other nervous system cancers in the US for men and women per year in 2014 was estimated to be 8.4 new cases per 100,000 per year. These rates are age adjusted and based on 2010–2014 cases and deaths. If we use the data from the interphone study, we can estimate that this number would increase by about a factor of 1.4 to about 11.8/100,000 for brain tumors among the heaviest cell phone users. This number might be compared to the number of traffic fatalities of 10.92 per 100,000 population per year in the US in 2015 (NHTSA). There are also studies that show effects such as increases in the incidence of loss sleep, lack of concentration, fatigue, loss of memory, etc. for exposures to low levels of RF fields (28, 29). The question can be, how important are these effects?

## Some theory with respect to mechanisms

A major problem in explaining the effects of weak EM fields has been that the quantum of energy in a single radio frequency photon is very small as compared to the thermal noise energy in the system or the energy required to break chemical bonds. In addition to the conservation of energy, the conservation of momentum is required along with the Pauli Exclusion Principle that no two Fermions can have the same quantum numbers in the same space. For coherent radical pairs, with parallel spins in the same orbit, recombination is forbidden and happen rapidly when the spins are antiparallel. This leads to the ability to control radical recombination rates using quantum selection rules and changing magnetic fields. Experimentally it has been shown that magnetic fields can change chemical reaction rates with static, low frequency and RF magnetic fields by more than 25% (18, 30, 31). Variation in the static magnetic fields can both increase and decrease the energy separation between triplet states of radicals and thus the frequency for transferring electrons or nuclei between energy levels that are actively involved in a chemical reaction (32). The long relaxation times for nuclear spins and the corresponding rapid variations in frequency responses can lead to changes in HT-1080 fibrosarcoma cell growth rates with variations in frequency as little as half a cycle per second at 16.5Hz for 4-day exposures to 9.8 μT in a static field of 45 μT (33). At microwave frequencies the mechanisms by which magnetic fields may modify chemical reaction rates needs to be explored. However, it is likely that there will be molecular energy level separations with different magnetic moments corresponding to these frequencies that modify the chemical reaction rates just as they do at infrared, optical, and lower radio frequencies.

Panagopoulos et al. (34) point out the importance of coherence and the polarization of the electric fields and their difference from naturally occurring fields in the possibilities of affecting ion transport through membrane channels with fields as weak as 4 ×10^−4^ V/m for pulses with repetition rates at frequencies corresponding to natural oscillating frequencies. The importance of polarization for magnetic fields is also shown in the paper by Gurhan et al. (10).

Recent data measured in our lab at the University of Colorado Boulder suggest that small changes in carrier frequency and modulation can make significant differences in the biological system responses, especially regarding the concentrations of biological signaling molecules such as calcium and hydrogen peroxide. Research needs to be done to show what low-level RF field modulation and exposure characteristics lead to biological effects, including changes in oxidative stress, how adaptive responses compensate for them and how they lead to damaging changes in cell function and health. It is known that resetting ROS and/or RNS baseline concentrations affect aging, cancers, and Alzheimer's (19). This has implication for how regulations might be established.

Biological systems contain many process functions that are comparable to those found in most electronic control systems (amplifiers, feedback loops, time delays, oscillators, comparators, noise generators, etc.). Many biological systems appear to stabilize their function with control loops that that can be modeled as amplifiers with negative feedback that occurs with a time delay (35). The timing or phase of an external perturbation to an oscillating system can either increase or decrease the amplitude of the oscillation. For example, if you push a swing at the top, you increase the amplitude and if you push at the bottom in the same direction, you decrease it. Therefore, the timing of an electromagnetic pulse with respect to oscillating biological processes can lead to either positive or negative effects (36). More work in this area is needed to explain how external EM fields alter basic biological processes.

## Some general observations about setting regulatory guidelines

Electric and magnetic fields serve many functions, from driving motors and lighting cities to digital computing and telecommunications. Harnessing EM fields has helped to make our civilization function as well as it does. Safety mechanisms and regulations were implemented to reduce the level of injury and death cause by the evolving electric grid and the emission of high-power radio waves by radio and TV stations, and radar. Current IEEE and FCC guideline protect against large amplitude/high energy EM fields that could lead to shocks and burns. The current limits at low frequencies are 5,000 V/m for exposures in air to prevent the firing of a nerve. Typically, only a small fraction of the external EMFs gets coupled into the body. On the other hand, the firing rate of nerve cells can be modified by weak electromagnetic fields and fields as low as 20 mV/m across the cell membranes have been shown to modify the firing rates in neonatal bovine fibroblast cells (37). Between these two extremes, the question arises: “Under what conditions are weak EM fields hazardous to humans and how can exposure to such fields be managed to minimize the potential harm?” The recent research suggests that the effects of low-level EM fields on living organisms are non-trivial and potentially harmful. Given these revelations; regulators, providers, and users are under pressure to reach agreement on the most reasonable approach to minimize potentially harmful effects.

There are several learning experiences from prior regulatory efforts that are instructive on how we might regulate the cellular telephone system. Regulatory efforts with things such as automobiles, nicotine products, medically beneficial narcotics, firearms, and public health responses to pandemics took a substantial amount of time, effort, and expense to formulate and deploy. Regulatory actions in all these areas were met with considerable political resistance, irrespective of the benefits to society. Large public information and education programs were required to formulate and implement even the most sensible requirements. Even when highly beneficial regulatory actions were deployed, large segments of the population ignored warnings and formal regulatory governance (e.g., - seat belts and speed limits in the auto industry). Industry is generally responsive to regulation when it can participate in the formulation processes. However, this is not necessarily true when it comes to end user/consumer communities. Many people tend to exercise their constitutionally guaranteed right to act with indifference and unbridled self-interest.

Safety and operational specifications that dictate how manufacturers design and construct products can help prevent injuries and fatalities. Government imposed rules for use are harder to implement and enforce. Education, information, and advisory warnings are not as difficult to implement, but often less effective. The ultimate outcomes for human health and safety rely heavily on user behavior and attitude which are highly variable and unpredictable.

## Some specific ideas for cellular telephony systems

In cell phones, WiFi communications devices, and cellular communications infrastructure, tradeoffs between system functionality and cost generally govern device and system designs and specifications. Adding / changing regulatory requirements are likely to have an impact on system infrastructure, revenue, and operating cost. A suitable balance must be struck between the two. The primary issues are:

1. What new limitations on RF exposure (exposure time, frequency, exposure levels) need to be imposed?
2. How can they be implemented and enforced?
3. How much will it cost to implement them?
4. What will be the economic and social impact?

Manufacturers would like to have standards that apply worldwide to maintain market rationality and competitive growth. Gaining worldwide acceptance of a common set of regulatory requirements is problematic. New regulations could significantly affect world-wide market size, growth, and cost. Defining and implementing new standards for the world's cellular telephony system is likely to become a political controversy that is argued for decades. Any significant changes will certainly increase user costs.

Four strategies for reducing RF exposure might include:

1. Designing the transmitter in user devices to reduce the instantaneous RF power levels emitted is the most direct way to reduce RF exposure but this has serious system performance implications and high implementation costs. Manufacturers already try to minimize the instantaneous RF power output of cell phones to maximize battery life. Further reductions in RF power output will significantly impact signal to noise ratio and reduce the maximum range of any given phone within a cell phone tower matrix. This will have a significant impact on system infrastructure (number of cell phone towers needed to provide coverage) and operating cost. Another technique for reducing instantaneous power output absorbed by users is to use narrow beam directional antennas in user devices that focuses the output power on the closest cell tower receiver. This approach is being incorporated into 5G systems. Size and weight considerations are a major constraint in mobile devices.
2. Reducing the density of RF power (Watts/M^2^) being absorbed by the body of a user can also be accomplished by increasing the distance between the transmitting antenna and the user's body and head. Power density falls off very rapidly with increasing distance from the transmitting antenna typically as 1/R^n^ reduction where n is a number usually >1. It is to be noted that increasing use of data and moving a smart phone away from the body reduces the power levels incident on the head.
3. Research data indicates biological effects of RF signals are frequency dependent. Redesigning transmitters to eliminate frequencies that are proven to produce biological effects could be employed, but this could have a significant impact on system capacity since each frequency band carries a lot of data in today's system.
4. The last, and least expensive approach is to limit cumulative user exposure to RF energy in a given period of time by shutting the phone “Off”. Establishing limits for the maximum accumulated duration of an individual's exposure would require extensive clinical testing on large populations of users. Once those numbers were set, there would be very little cost impact on the operators or the users to implement. Major issues would be lack of access in emergency situations and gaining user acceptance on such limitations and the self-discipline to avoid over exposure. To help with that applications software could be installed on the phone that calculates the accumulated exposure and then reports the data on the phone's visual readout.

A more stringent approach would be to have the phone's controller shut the phone off until the elapsed time between sessions had passed. A third approach would be to limit the types of long duration applications that could be accessed from hand-held devices (Gaming, streaming broadcasts, entertainment programs and other long duration feeds).

Another approach might be minimizing the number of times a cell phone provides location and ranging data to the cell system when it is not in use or moving. The phone's accelerometer and a GPS receiver could inform to the cellular system only when the phone's location changes or when the user attempts to make a call. The most important aspect of implementing controls over user behavior is education and information. User discipline over use is preferred. Enforcement of mandatory limitations on use would be as unworkable in the current US political environment as the mandatory use of masks during the most recent COVID-19 Pandemic.

These suggested changes do not solve the problems for people who have problems from exposures to low levels of EM fields that are outside their control such as an increase in reactive oxygen levels or having problems sleeping after the installation of a base station in their neighborhoods.

## Conclusion

The data above and many more papers not cited indicate that biological systems can sense and respond to very weak electric and magnetic field by changing biological parameters such as reactive oxygen species concentrations at the cellular level which affect health and wellbeing of living organisms. High concentrations of reactive oxygen species for extended periods of time are known to be associated with adverse health effects (19). There are also many cases where no damaging effects have been observed. It is presumed that the body's feedback and repair systems keep the concentrations of these molecules within the normal operating ranges and the cumulative effects of RF energy are negligible. We hypothesize that EM effects vary from person to person and are a function of exposure conditions in conjunction with other stresses that affect concentrations of these molecules. Note this degree of variability explains why many papers do not show EM effects while some of the experiments on hypersensitive people show effects. We have chosen not to go into discussion of hypersensitive people as it would take more space than we wish to devote to it in this paper.

It is clear that more research needs to be done to enable definition of standards for RF exposure that are reasonable and allow a simple, low-cost communications system to function safely. Although both industry and government have funded significant amounts of expensive research, relatively few studies have used radical pair theory and other quantum mechanical models to guide their experiments or track the chemical changes induced by exposures to weak electromagnetic fields. Additionally, they have not delt with long term effects of exposure to low-level exposure that take into account biological feedback and repair systems, that may not be able to handle the effects of compounding stresses and the fact that humans have different responses at different time.

Forcing a solution that eliminates all wireless communications is not a reasonable approach. Allowing the telecommunications industry and users to ignore the potential harm indicated by some of the experiments showing the effects of weak field exposures is equally unsatisfactory given the data that are currently available. Imposing operating standards without understanding the root causes in science, and social impacts and costs is tempting, but also potentially dangerous and can potentially lead to health problems for a large fraction of the population.

In the US, most industries can be held liable for not pursuing research on the safety of their products. With such a large number of users, it is incumbent on system designers, operators, managers, and regulators to invest the time and energy to understand the risks of long-term exposure to low-level EM fields to determine potential health hazards. In the short term, implementing ways to reduce exposure voluntarily is likely to be the cheapest solution, but human behavior is often unpredictable and unreliable. Ultimately more research will better define the conditions where EM exposures can lead to changes in the biological system that are not compensated by biological control systems and repair mechanisms.

## Data availability statement

The original contributions presented are available in the references or by contacting the corresponding author.

## Author contributions

Both authors listed have made a substantial, direct, and intellectual contribution to the work and approved it for publication.

## Funding

This study received support from DARPA Grant No HR00111810006 and the Milheim Foundation for financial support that enabled much of our work over the past several years.

## Conflict of interest

The authors declare that the research was conducted in the absence of any commercial or financial relationships that could be construed as a potential conflict of interest.

## Publisher's note

All claims expressed in this article are solely those of the authors and do not necessarily represent those of their affiliated organizations, or those of the publisher, the editors and the reviewers. Any product that may be evaluated in this article, or claim that may be made by its manufacturer, is not guaranteed or endorsed by the publisher.
